# Drop finger caused by lung cancer metastasis

**DOI:** 10.1002/rcr2.1280

**Published:** 2024-01-18

**Authors:** Toshiyuki Sumi, Jun Sakakibara‐Konishi, Keito Suzuki, Hirofumi Chiba

**Affiliations:** ^1^ Department of Pulmonary Medicine Hakodate Goryoukaku Hospital Hakodate Japan; ^2^ Department of Respiratory Medicine and Allergology Sapporo Medical University School of Medicine Sapporo Japan; ^3^ Department of Respiratory Medicine, Faculty of Medicine Hokkaido University Sapporo Japan

**Keywords:** drop finger, lung cancer, malignant tumour metastasis, MET exon 14 skipping mutation, tumour‐induced nerve compression

## Abstract

Skeletal muscle metastasis of lung cancer is rare. However, clinicians should be aware that tumour‐induced nerve compression symptoms may develop.

## Graphical abstract

We present a rare case of posterior interosseous nerve (PIN) palsy secondary to lung cancer metastasis.

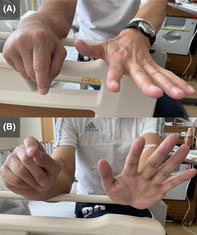

## CLINICAL IMAGE

**FIGURE 1:**
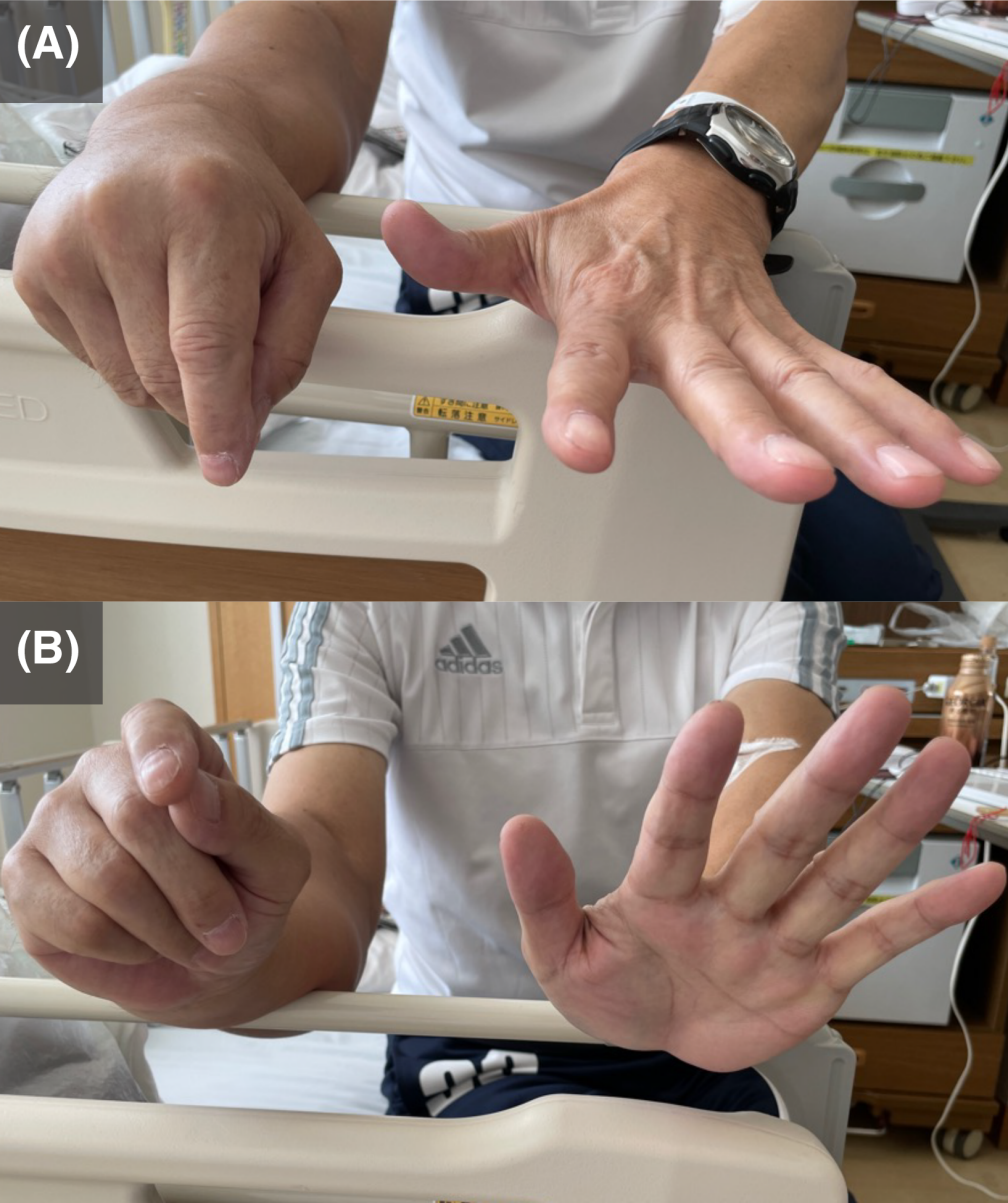
Drop finger. (A) Extension position of the wrist and fingers. The extension of the right fingers is impaired. (B) Extension of the fingers and dorsiflexion of the wrist. Dorsiflexion of the right wrist is possible, although the extension of the fingers is impaired.

**FIGURE 2:**
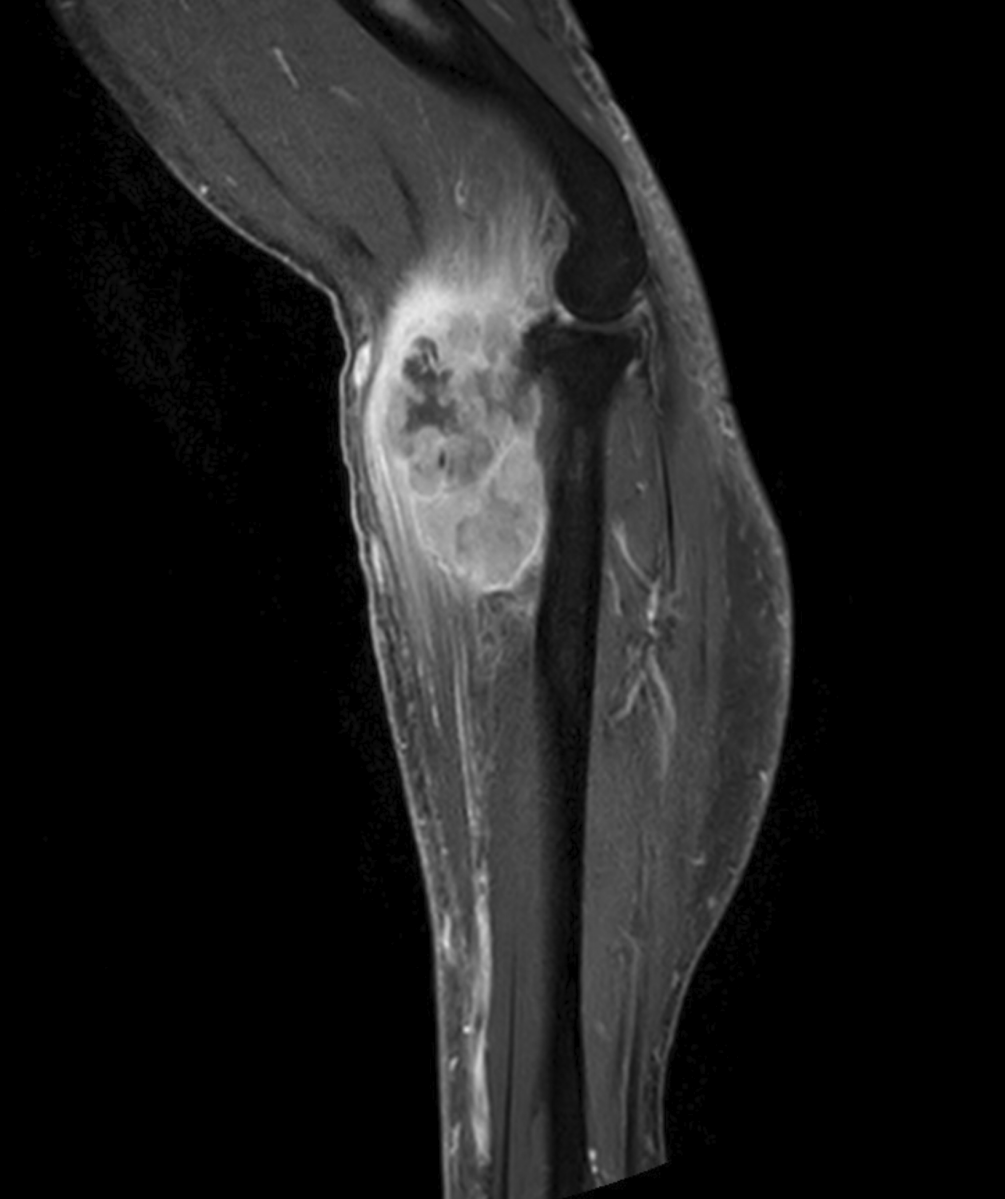
Magnetic resonance imaging findings of the right forearm. Massive lesion (9 cm × 4 cm) near the radius with suspected posterior interosseous nerve invasion.

**FIGURE 3:**
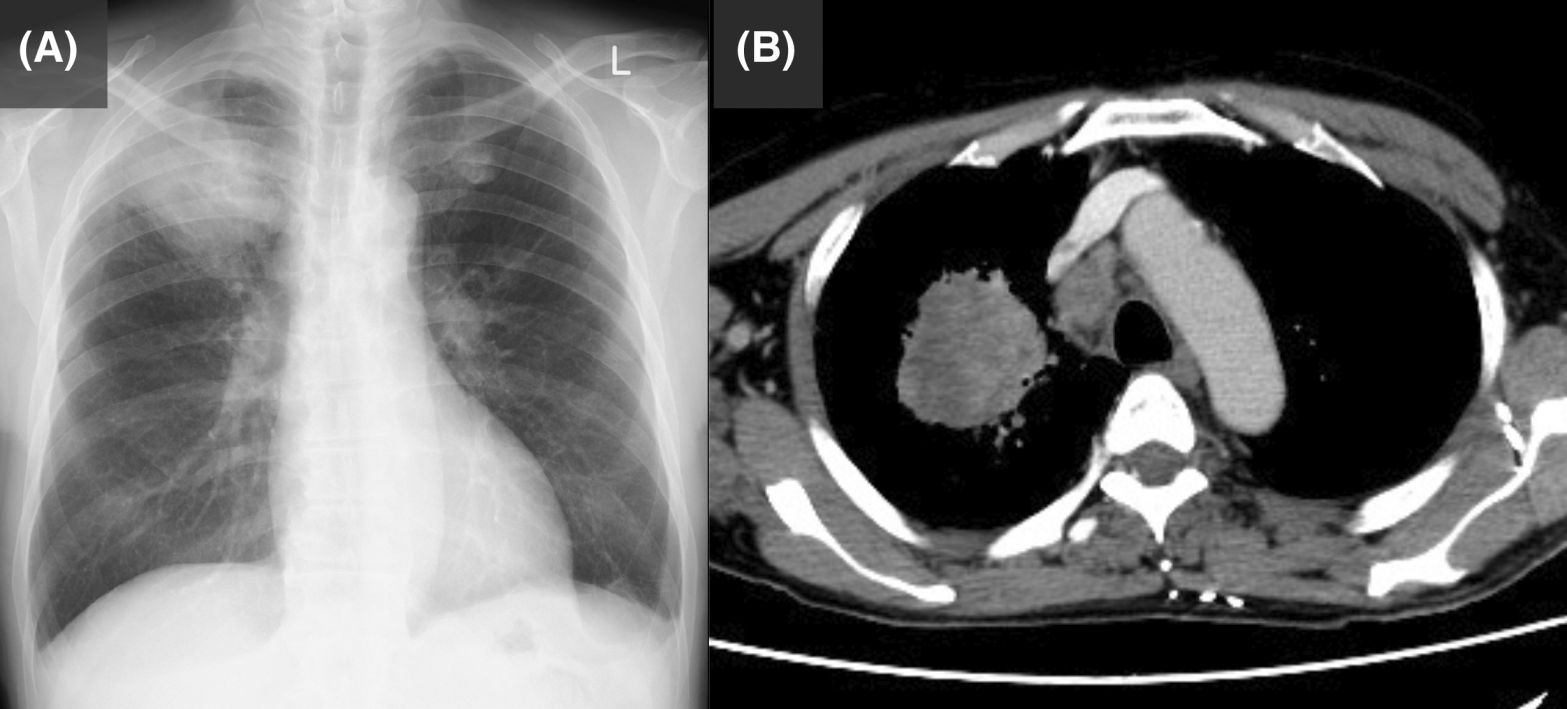
x‐Ray and enhanced computed tomography of the chest. (A) A large tumour is observed in the right upper lung field in the x‐ray. (B) Chest enhanced computed tomography showing a tumour lesion in the right upper lobe and enlargement of the mediastinal lymph node #4R.

A 65‐year‐old man presented with right forearm pain and paralysis of his right‐hand fingers. Physical examination showed right forearm swelling and drop finger with no sensory deficits in the fingers (Figure 1). Magnetic resonance imaging revealed a forearm mass (Figure 2), whereas chest x‐ray and computed tomography identified a right upper lobe mass (Figure 3) and right renal tumour; all masses were histologically identified as non‐small cell lung cancer. Consequently, posterior interosseous nerve (PIN) palsy secondary to lung cancer metastasis was diagnosed. The patient tested positive for MET exon 14 skipping mutation. Palliative radiation therapy (30 Gy in 10 fractions) was administered to the forearm tumour, followed by systemic tepotinib therapy. The drop finger showed no improvement despite tumour size reduction.

PIN palsy is commonly caused non‐traumatically by mesenchymal tumours like lipomas, inflammation, or nerve entrapment due to anatomical abnormalities.^1^, ^2^ Periosteal lipoma is the most frequent solid tumour near the proximal radius;^2^ however, palsy due to tumour metastasis is rare. Compressive PIN palsies typically warrant surgical intervention.^1^ However, we opted for palliative radiation to alleviate pain given the malignant nature and potential tissue invasion risk. While skeletal muscle metastasis from lung cancer is uncommon, tumour‐induced nerve compression symptoms can occur.

## AUTHOR CONTRIBUTIONS

Toshiyuki Sumi conceived the idea for the manuscript and drafted it. Jun Sakakibara‐Konishi and Keito Suzuki contributed to the clinical management of the patient. Hirofumi Chiba revised the manuscript for intellectual content. All authors contributed to and approved the final version of the manuscript.

## CONFLICT OF INTEREST STATEMENT

None declared.

## ETHICS STATEMENT

The authors declare that appropriate written informed consent was obtained for the publication of this manuscript and accompanying images.

## Data Availability

The data that support the findings of this study are available from the corresponding author upon reasonable request.
